# Filling Teeth

**Published:** 1899-03

**Authors:** L. L. Barbour

**Affiliations:** Toledo, Ohio


### Filling Teeth.

BY L. L. BARBOUR, D.D.S., TOLEDO, OHIO.

The treatment of a cavity of decay by filling must have a two-fold object in order to subserve its best purposes: First, the restoration of the affected part to a healthy condition; and second, the prevention, as far as possible, of a recurrence of the lesion.

It is only the second that I will attempt to speak of, because it is the contouring of fillings to which I direct your attention at this time.

While the simple filling of a cavity, if properly done, will generally prevent the extension of decay on exposed surfaces, the same operation on surfaces less favorably situated may utterly fail to subserve the desired end. The contiguity of the proximal surfaces of the teeth greatly favors the retention of food and the harboring of micro-organisms, while at the same time it prevents the free use of any cleansing methods upon such surfaces.

For these reasons proximal surfaces of teeth are attacked by caries more frequently than many others. When once affected by caries their restoration by filling is more difficult owing to their inaccessibility, and while the operations on this account often lack the perfection that would otherwise be secured and the fillings consequently fail, the recurrence of decay is more largely due to the same influence that at first brought it about. This being the case, it is obvious that the original conditions must be changed if immunity from decay is to be expected. And with this end in view the process of filing or cutting the proximal surfaces so as to free them from contact has been practiced on the principle of "no contact, no decay." This was, however, largely abandoned, but in about 1870 it was renewed in a modified form by the teachings of Dr. Robert Arthur, I think it was. This method consisted of so changing the proximal surfaces that the point of contact should be near or quite at the cervical margin, but this was not correct in principle and it soon went out of general practice.

The failure to secure freedom from decay by an unnatural alteration of the surfaces leads to a more careful investigation and the gradual adoption of more rational and scientific methods for its prevention, among which is the protection and maintainance of the gum tissue in the inter-proximal space. In the normal contact of the teeth the gum tissue usually fills the inter-proximal space, but when decay takes place on the proximal surface and the teeth begin to drop together, the gum is forced out of the space and the arched form is destroyed, and in extensive decay there is little or no gum left midway between the teeth.

The proper management of these cases consists in wedging the teeth apart to their original position by any method you may desire, so long as you do not destroy the arched form of gum tissue, and building the fillings into such contours that they will be retained. The form and density of the contact point on proximal fillings have much to do with the proper maintainance of the space, which in turn, is one of the principal features in the prevention of recurrent decay. The contact should be small and somewhat rounded. If the surface is broad and flat, particles of food, such as shreds of meat, are held between the teeth to decompose.

Some operators are in the habit of making broad, flat contacts built firmly against the proximating teeth. The error in this lies in the fact that no two teeth can be built so closely together that food will not, at some time in mastication, be forced between them. Fillings at the contact points should be made as dense as possible in order to maintain their form against friction in the movement of one tooth against another.

The fillings should be well seated, because if a large seating capacity is given we do not have to depend so much upon deep under-cuts, which are necessarily hard to fill. The buccal and lingual outline of the filling should be so as not to allow any of the tooth tissue to touch its fellow. The filling should also, in nearly all cases, extend cervically far enough to be covered by the gum tissue, which, if left in a healthy condition and proper form, will come as nearly preventing a recurrence of decay at that point as anything can. The cervical margin, or seat of cavity, should be rounded slightly, but still with a dipping toward the denture or body of tooth. The mesio-lingual and mesio-buccal cusp (if an anterior cavity) should not be left too high, making the filling to concave from side to side. This, with the proper finishing, will make about as good a defense against the recurrence of decay upon the proximal surfaces of teeth as it is possible to make.

